# Diversity Analysis of Bacterial and Function Prediction in Hurunge From Mongolia

**DOI:** 10.3389/fnut.2022.835123

**Published:** 2022-03-25

**Authors:** Wuyundalai Bao, Yuxing He, Wei Liu

**Affiliations:** College of Food Science and Engineering, Inner Mongolia Agricultural University, Hohhot, China

**Keywords:** high-throughput sequencing, Mongolia, Hurunge, bacterial diversity, functional prediction

## Abstract

With the continuous infiltration of industrialization and modern lifestyle into pastoral areas, the types and processing capacity of Hurunge are decreasing, and the beneficial microbial resources contained in it are gradually disappearing. The preservation and processing of Hurunge are very important for herdsmen to successfully produce high-quality koumiss in the second year. Therefore, in this study, 12 precious Hurunge samples collected from Bulgan Province, Ovorkhangay Province, Arkhangay Province, and Tov Province of Mongolia were sequenced based on the V3–V4 region of the 16S rRNA gene, and the bacterial diversity and function were predicted and analyzed. There were significant differences in the species and abundance of bacteria in Hurunge from different regions and different production methods (*p* < 0.05). Compared with the traditional fermentation methods, the OTU level of Hurunge fermented in the capsule was low, the *Acetobacter* content was high and the bacterial diversity was low. *Firmicutes* and *Lactobacillus* were the dominant phylum and genus of 12 samples, respectively. The sample QHA contained *Komagataeibacter* with the potential ability to produce bacterial nanocellulose, and the abundance of *Lactococcus* in the Tov Province (Z) was significantly higher than that in the other three regions. Functional prediction analysis showed that genes related to the metabolism of bacterial growth and reproduction, especially carbohydrate and amino acid metabolism, played a dominant role in microorganisms. In summary, it is of great significance to further explore the bacterial diversity of Hurunge for the future development and research of beneficial microbial resources, promotion, and protection of the traditional ethnic dairy products.

## Introduction

Traditional fermented koumiss (airag or chigee) is fresh horse milk without high-temperature sterilization in wooden buckets, porcelain cans, or animal skins (1), koumiss at room temperature for 1–3 days with natural microbial starter retained in the previous year or batch of koumiss, the whole process constantly stirs the fermented koumiss with wooden sticks and constantly adds fresh horse milk, in order to remove carbon dioxide and ensure uniform fermentation. Accelerate natural fermentation to eliminate the reproduction of pathogens (2). Koumiss is mainly fermented by microorganisms such as lactic acid bacteria and yeast (3), which can produce lactic acid, alcohol, and other small molecular flavor substances, and is rich in essential amino acids, trace elements, and various vitamins. Therefore, it has a unique flavor, texture, acidity, and health benefits (4–6). For hundreds of years, koumiss has been regarded as not only a kind of food, but also a natural alternative medicine, so nomads invented “koumiss therapy,” which combines traditional Mongolian medicine with koumiss used in the clinical treatment of intestinal indigestion, hypertension, tuberculosis, and other cases (7, 8). Therefore, koumiss is considered to be a complete diet, which is rich in nutrients. The nomads on the grasslands of Central Asia and Mongolia can persist in the nomadic lifestyle and cold grassland climate without the rich nutrients of koumiss. They constitute an important part of the daily diet of Mongolian herdsmen (9–11).

It has always been a Mongolian custom to use the starter to koumiss, the native starter used to make traditional fermented koumiss is called Hurunge. Since ancient times, the traditional method of fermenting koumiss is to use the skin made of cowhide to dry the fermented koumiss in the previous year and can continue to be used to ferment koumiss after putting fresh horse milk in the summer of the second year. Dried skins can well protect the activity of the starter. The Hurunge is fermented koumiss in the previous year into millet or raisins above 1 kg, put it into a clean bag sealed preservation, the next year fermented koumiss, add a certain amount of Hurunge in the fresh horse milk and continue to stir with sticks, you can complete the fermentation so that the fermented koumiss will maintain a good taste and quality. Studies have shown that Hurunge contains a complex microbial community, which strongly affects the final microbial community and the resulting quality of koumiss (12). However, with the development of science and technology, the brewing of koumiss has developed from traditional methods to commercial starter brewing. The traditional manual brewing method is gradually disappearing, resulting in the loss of a large number of excellent strains.

Mongolia belongs to the temperate continental steppe climate, with a significant temperature difference between day and night, its forage species and quantity are high-quality green, and it is rich in nutrients, so there is no doubt about the quality of fresh horse milk (13). Bulgan Province, Ovorkhangay Province, Arkhangay Province, and Tov Province are the main production areas of traditional koumiss. It is necessary to collect precious samples of Hurunge and analyze the bacterial diversity in this area because the rich bacteria play an important role in the quality and flavor of Hurunge, and play an important role in improving the quality of commercial starter fermented koumiss in the future. However, the traditional microbial culture methods are time-consuming and laborious, and some microorganisms cannot be isolated depending on the existing isolation methods, so it is easy to ignore the overall microbial diversity (14). Therefore, it is often limited to using traditional methods to analyze microbial diversity. In recent years, the rapid development of sequence technology, such as the second-generation sequencing platform Illumina-Miseq, provides a convenient method for the analysis of microbial components in various samples. It can fully cover the complex and low abundance of microbial communities in the samples and analyze the diversity of microbial communities at the genus and even species level (15).

In this study, samples of Hurunge from four regions of Mongolia were collected, and a high-throughput sequencing technique was used to predict the composition and function of the bacterial community. These data are not only of great significance to further study the mining of probiotics in Hurunge samples in Mongolia, but also have important prospects for the commercialization and industrial production of Koumiss.

## Materials and Methods

### Collection of Samples

**Figure 1:**
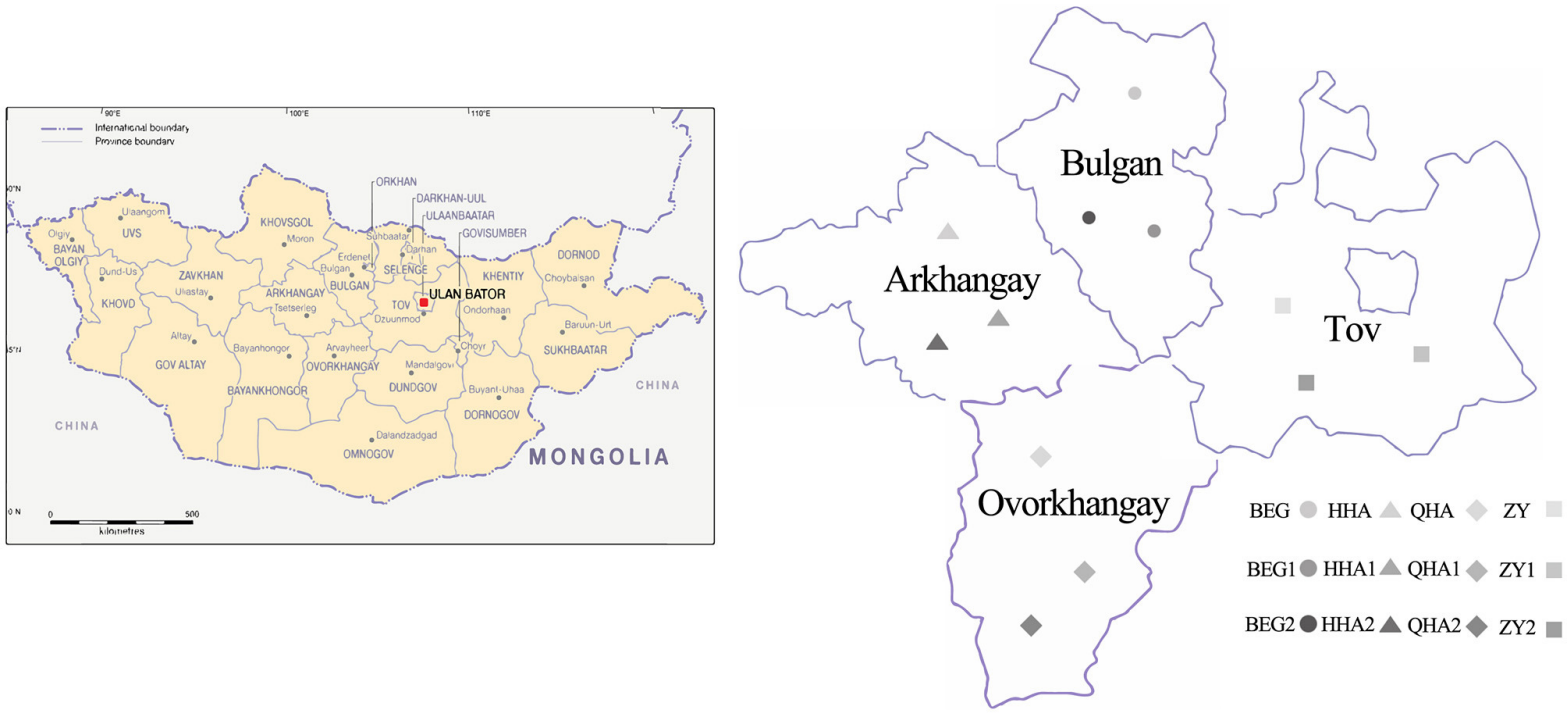
Distribution of 12 Hurunge samples in Mongolia.

We collected 12 samples of Hurunge from town in four regions of Mongolia: Bulgan Province, Ovorkhangay Province, Arkhangay Province, and Tov Province. All of them were brewed by herdsmen through natural fermentation and were used for the fermentation of koumiss. Samples BEG, BEG1, and BEG2 were collected from Burgan Province (B), samples HHA, HHA1, and HHA2 (H) were collected from Ovorkhangay Province, samples QHA, QHA1, and QHA2 were collected from Arkhangay Province (Q), and samples ZY, ZY1, and ZY2 were collected from Tov Province (Z). These sampling sites cover most of the main producing areas of “Hurunge” in Mongolia, as shown in Figure 1. The samples BEG, HHA, QHA, and ZY are fermented “Hurunge” in animal skins, and the rest are made by the traditional fermentation process. At the same time, the production technology, fermentation environment, fermentation time, and storage conditions of “Hurunge” samples were recorded. The collected samples are stored in a sterile centrifugal tube and transferred to the laboratory in the shortest possible time for a foam box containing dry ice.

### DNA Extraction and PCR Amplification

Microbial community genomic DNA was extracted from 12 samples using the E.Z.N.A.^®^ soil DNA Kit (Omega Bio-tek, Norcross, GA, US) according to the manufacturer's instructions. The DNA extract was checked on 1% agarose gel, and DNA concentration and purity were determined with NanoDrop 2000 UV-vis spectrophotometer (Thermo Scientific, Wilmington, USA). The hypervariable region V3–V4 of the bacterial 16S rRNA gene was amplified with primer pairs 338F (5′-ACTCCTACGGGAGGCAGCAG-3′) and 806R (5′-GGACTACHVGGGTWTCTAAT-3′) by an ABI GeneAmp^®^ 9700 PCR thermocycler (ABI, CA, USA). The PCR amplification of 16S rRNA gene was performed as follows: initial denaturation at 95°C for 3 min, followed by 27 cycles of denaturing at 95°C for 30 s, annealing at 55°C for 30 s and extension at 72°C for 45 s, and single extension at 72°C for 10 min, and end at 4°C. The PCR mixtures contain 5 × *TransStart* FastPfu buffer 4 μl, 2.5 mM dNTPs 2 μl, forward primer (5 μM) 0.8 μl, reverse primer (5 μM) 0.8 μl, *TransStart* FastPfu DNA Polymerase 0.4 μl, template DNA 10 ng, and finally ddH_2_O up to 20 μl. PCR reactions were performed in triplicate. The PCR product was extracted from 2% agarose gel and purified using the AxyPrep DNA Gel Extraction Kit (Axygen Biosciences, Union City, CA, USA) according to manufacturer's instructions and quantified using Quantus™ Fluorometer (Promega, USA).

### Illumina MiSeq Sequencing

Purified amplicons were pooled in equimolar and paired-end sequenced on an Illumina MiSeq PE300 platform/NovaSeq PE250 platform (Illumina, San Diego, USA) according to the standard protocols by Majorbio Bio-Pharm Technology Co. Ltd. (Shanghai, China). The raw reads were deposited into the NCBI Sequence Read Archive (SRA) database (Accession Number: PRJNA793521).

### Processing of Sequencing Data

The raw 16S rRNA gene sequencing reads were demultiplexed, quality-filtered by fastp version 0.20.0 (16), and merged by FLASH version 1.2.7 (17). Operational taxonomic units (OTUs) with 97% similarity cutoff (18) were clustered using UPARSE version 7.1 (19), and chimeric sequences were identified and removed. The taxonomy of each OTU representative sequence was analyzed by RDP Classifier version 2.2 (20) against the 16S rRNA database (e.g., Silva v138) using a confidence threshold of 0.7.

### Data Analysis

Mothur (v.1.30.2) software was used to calculate the Alpha diversity index of the flattened sample of OTU levels, such as Sobs, Chao1, Ace, Shannon, Simpson, and Coverage index. The Student's *t*-test method was used to test the significant difference between groups, and the R language tool was used to draw a dilution curve. The beta diversity distance matrix was calculated by Qiime software, and the Principal Coordinates Analysis (PCoA) diagram and relative abundance histogram of each sample gate (≥ 1%) and genus (≥ 1%) horizontal group were drawn by the R language (version3.3.1) tool. The prediction functional genomic analysis of the microbial community in the sample was carried out by using PICRUSt2 software, and the key functional gene modules of Pathwaylevel1, Pathwaylevel2, and Pathwaylevel3 metabolic pathway were selected from the KEGG functional module, and the R language tool was used to draw the relative abundance heat map of the functional module.

## Results

### Sample Sequence Richness and Alpha-Diversity

A total of 577,699 high-quality sequences of bacteria were produced in 12 samples of Hurunge, with an average length of 447 bp, and the length of high-quality sequences was concentrated in 441–460 bp. After sequencing at a 97% similarity level, 141 OTUs were identified from 12 samples of Hurunge.

**Figure 2:**
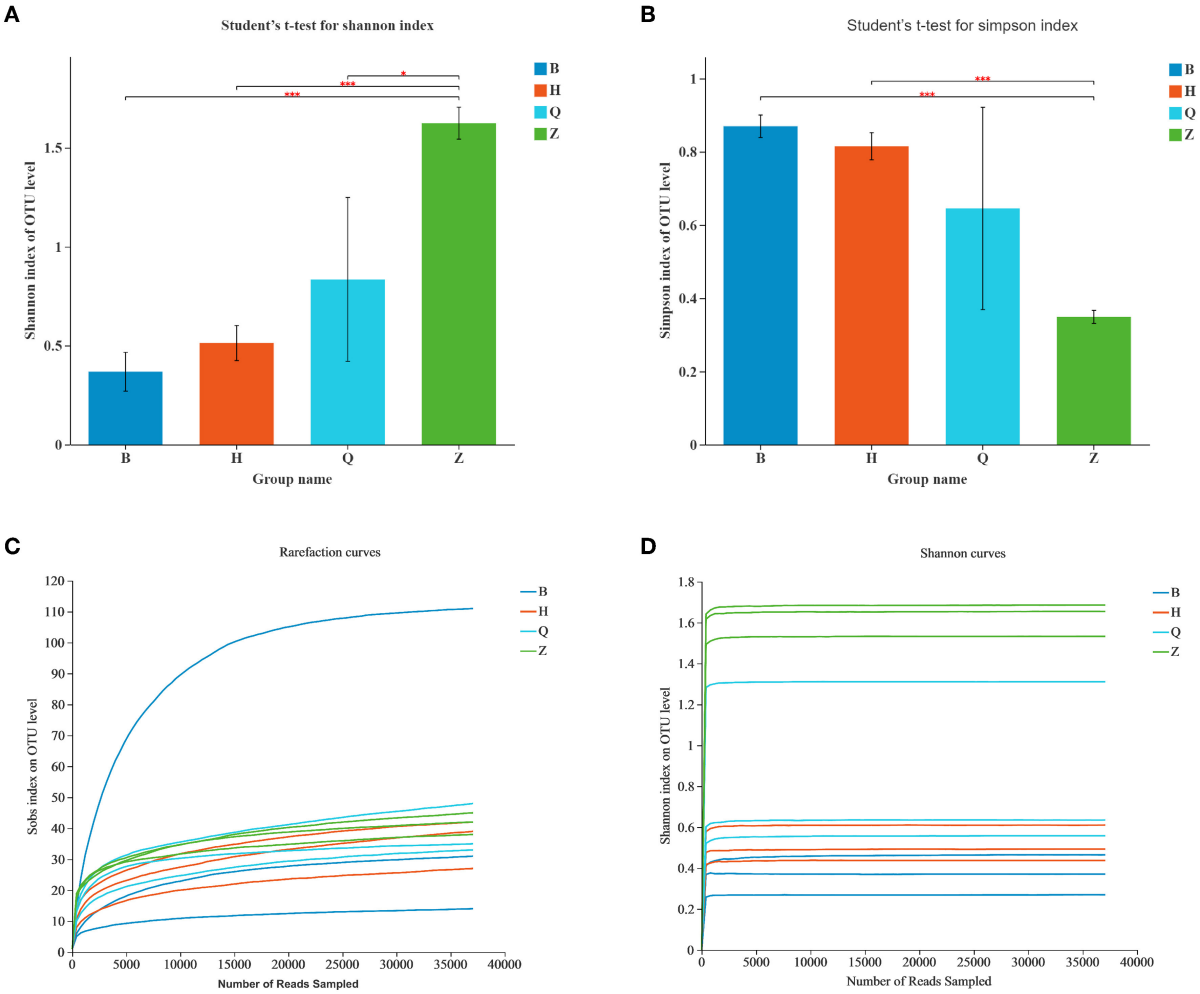
Alpha-diversity of the bacterial community in Hurunge samples. **(A)** The bacterial diversity is estimated by the Shannon index. **(B)** The bacterial diversity is estimated by the Simpson index. **(C)** Sob index curve of each sample and **(D)** Shannon index curve.

Alpha diversity can reflect the richness and diversity of the microbial community. Chao1 index, Ace index, Simpson index, and Shannon index are obtained by Alpha diversity analysis. Chao1 and Ace index are often used to estimate the total number and richness of species and are used to evaluate whether a sample has this species or not. The higher the index value, the more species, and species. Simpson and Shannon indexes are often used to quantitatively describe the biological diversity in a certain region, and more consideration is given to the evenness of species distribution. The smaller the Simpson index value is, the larger the Shannon index value is, indicating that the community diversity is higher. According to the data in Table 1, the total number of bacterial species in sample BEG1 is the most, while the number of BEG species is the least. The bacterial community diversity of sample ZY2 is the highest and that of BEG2 is the lowest. In general, the bacterial diversity in the Z area is higher than that in other places, as shown in Figures 2A,B.

**Table 1:** Sequence information and diversity value of 12 Hurunge samples.

**Areas**	**Sample-name**	**Sequences**	**Ace**	**Chao**	**Shannon**	**Simpson**	**Sobs**	**Coverage**
B	BEG	37,312	16.45	15.50	0.37	0.84	14	0.9999
	BEG1	41,367	111.80	111.86	0.47	0.87	111	0.9999
	BEG2	37,076	33.45	36.00	0.27	0.90	31	0.9999
H	HHA	47,421	34.85	34.00	0.49	0.80	29	0.9999
	HHA1	57,819	47.37	45.60	0.61	0.79	45	0.9999
	HHA2	48,399	52.32	48.50	0.44	0.86	41	0.9998
Q	QHA	54,858	33.85	33.25	1.31	0.33	33	1.0000
	QHA1	57,213	59.40	56.43	0.63	0.79	50	0.9998
	QHA2	53,615	36.58	35.25	0.56	0.82	35	1.0000
Z	ZY	40,703	45.94	43.75	1.53	0.33	40	0.9999
	ZY1	40,155	49.59	49.20	1.65	0.36	45	0.9998
	ZY2	42,188	47.33	50.50	1.69	0.36	43	0.9999

The dilution curve and aroma concentration curve were drawn according to the Sob index and Shannon index, and then the sequence quality and depth were evaluated. As shown in the Sob index curve in Figure 2C, the number of bacterial OTUs in the sample increased with the increase of sequencing depth. Figure 2D showed that the Shannon curve reached a stable platform, and the Good's coverage value was higher than 0.99, indicating that at the current sequence depth, diversity of the most bacteria in the sample was captured, enough to analyze most of the microflora (21). The current sequence quantity can meet the requirements of subsequent bioinformatics analysis.

### Comparison of Bacterial Communities Among Groups

**Figure 3:**
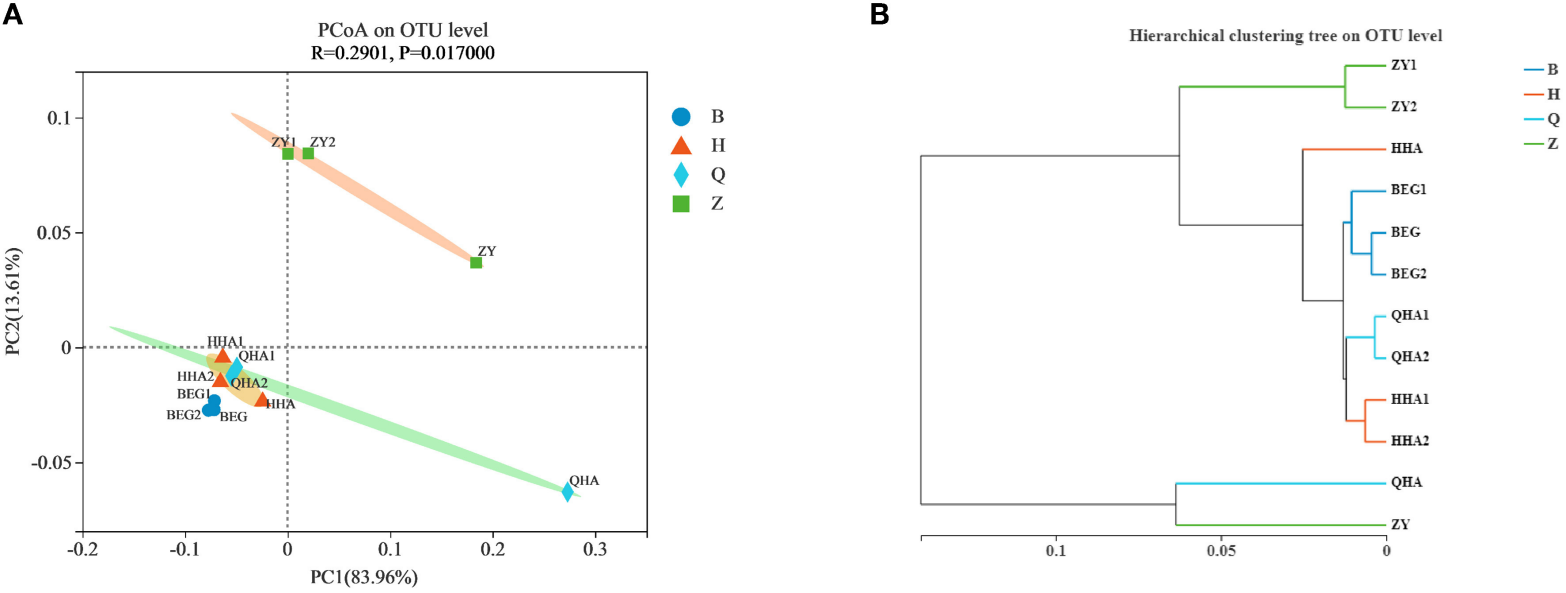
**(A)** PCoA analysis and **(B)** sample level cluster analysis of the bacterial community in koumiss samples at OTU level.

Although the original materials of 12 Hurunge samples were fresh horse milk and no commercial starter was added in the production process, the composition of the bacterial community of 12 Hurunge samples was different due to various reasons such as the production and preservation process, fermentation container, fermentation temperature, regional environment, and microbial species of milk source. Therefore, in order to explore the composition and difference of bacterial structure in different samples, the PCoA diagram of bacterial community principal coordinates is drawn based on weighted UniFrac to study the similarity or difference of sample community composition. It can be seen from Figure 3A that the three Hurunge samples from area Z are far away from other samples in the figure, indicating that the bacterial community structure and composition in area Z are significantly different from those in other areas (*P* < 0.05), while the other three areas are very close. The points represented by the samples in areas B, H, and Q are close in the spatial distribution distance and basically distributed in the same area. It shows that there is no significant difference in bacterial community composition of Hurunge samples from these three regions. However, sample QHA is far away from other samples and is alone in a region.

Hurunge may have specific connections or differences in the microbial community structure due to differences in collection location, production habits, and containers. In order to further analyze the significance of the dominant microflora in Hurunge and the distribution of microorganisms in the collection site, the sample hierarchical cluster analysis of the distance matrix of bacterial community Figure 3B was carried out, which was consistent with the result of PCoA diagram. At the OTU level, the bacterial community could be divided into different groups, indicating that there were significant differences in bacterial species and richness with different geographical locations of the samples collected(*p* < 0.05). The QHA samples and ZY samples in the bacterial community are grouped together, and the sample HHA is also in a single branch. The 3 samples are all from Hurunge fermented and preserved in animal skins. Therefore, it is inferred that the fermentation environment is similar and the microbial species contained in the samples are also similar.

### Classification and Composition of Bacterial Communities

**Figure 4:**
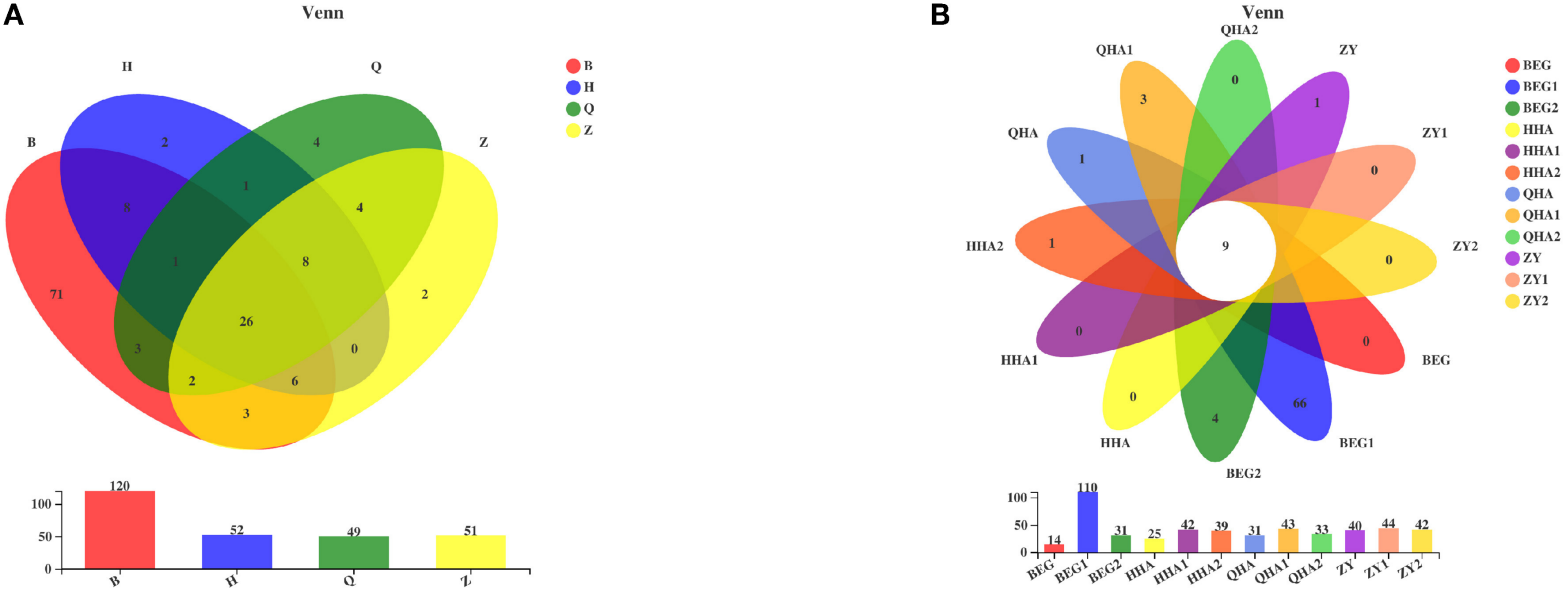
Venn diagrams. **(A)** Venn diagram of OTU in 4 places of Mongolia is analyzed according to bacterial microbial diversity. **(B)** Venn diagram of OTU of each sample is analyzed according to bacterial microbial diversity.

Venn diagram can reflect the situation of common and unique species among different groups. In this study, a Venn diagram was used to compare and analyze the bacteria in Hurunge samples at the OTU level. The results showed that there were significant differences among groups. At the OTU level, as shown in Figure 4A, 120, 52, 49, and 51 OTUs were obtained from the samples in regions B, H, Q, and Z, respectively, of which 26 OTUs were shared by Hurunge samples in 4 regions. The sample in region B had the highest OTU level, reaching 120, which may be related to its unique geographical environment. In addition, as shown in Figure 4B, 14, 25, 31, and 40 OTUs were detected in samples BEG, HHA, QHA, and ZY, respectively. These four were from Hurunge fermented and preserved in animal skins. Compared with the traditional fermentation method, the OTU level was lower, and only 9 OTUs of 12 samples were common.

**Figure 5:**
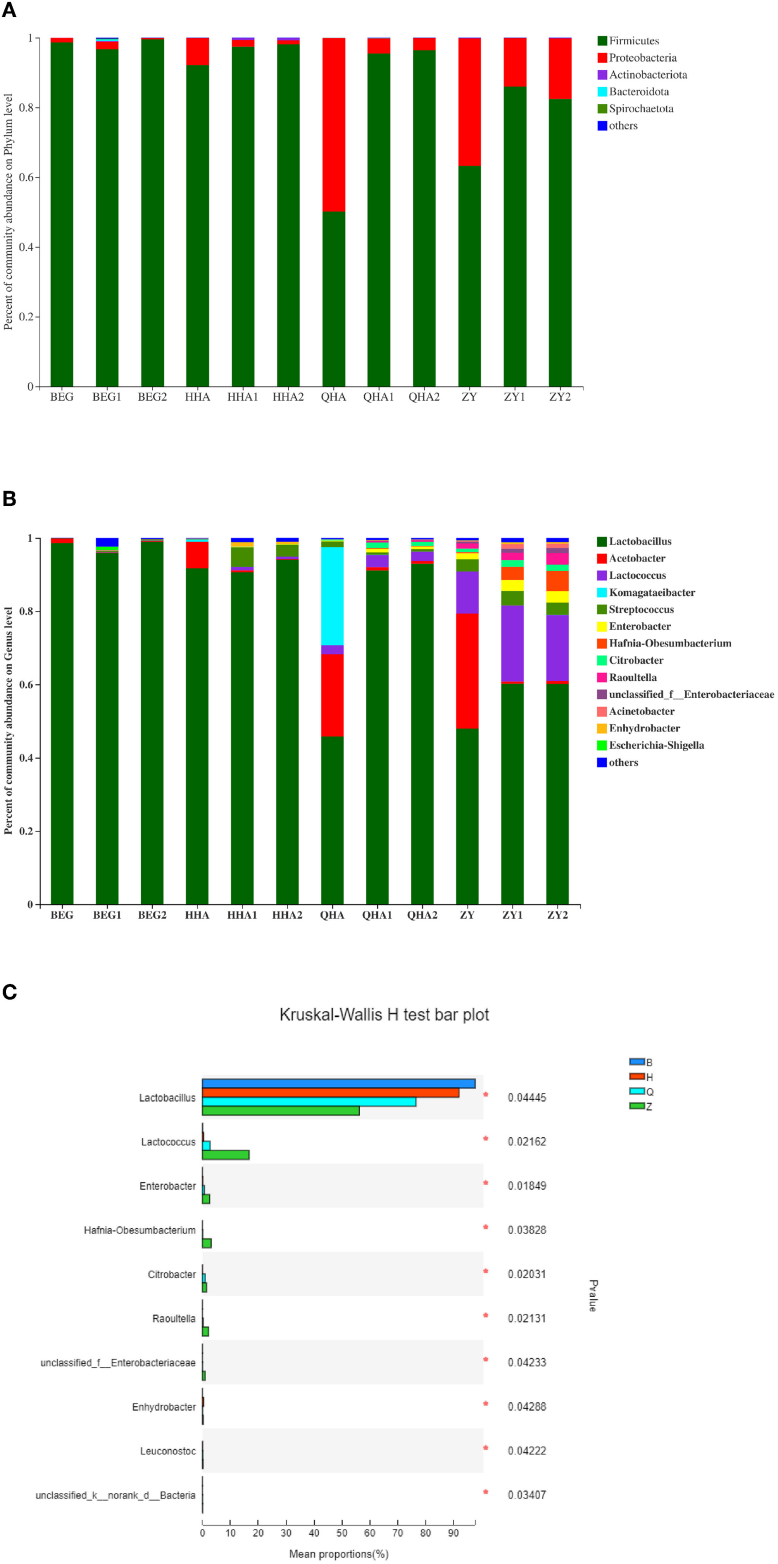
Bacterial community structure of different Hurunge samples. **(A)** Phylum level and **(B)** Genus level. **(C)** Relative abundance at genus level with the significant difference among samples from different regions. The proportion of which is less than 1% is not listed.

Through 16S rRNA Gene Sequencing analysis, 12 samples of koumiss were identified with 17 phyla, 98 genera, and 120 species. The relative abundance of bacterial communities at the phylum and genus level is shown in Figure 5A. A total of 12 samples of Hurunge containing 5 main bacterial phyla (relative content > 1%), namely *Firmicutes, Proteobacteria, Actinobacteria, Bacteroidetes*, and *Spirochaetota*, which is consistent with the previous survey of bacterial diversity in koumiss (22). *Firmicutes* is the dominant bacteria in all samples, with the exception of QHA and ZY, the content of *Firmicutes* in other samples is more than 80%. The proportions of Firmicutes in samples QHA and ZY were 50.1 and 63.3%, respectively. Compared with other samples, Proteobacteria was the second dominant phylum.

As can be seen from Figures 5B,C, the main genera in the 4 regions of Mongolia are *Lactobacillus, Acetobacter, Lactococcus, Komagataeibacter, Streptococcus, Enterobacter, Hafnia-Obesumbacterium, Citrobacter, Raouitella, Acinetobacter, Escherichia-Shigella*, and other 11 genera with a relative content of more than 1%. The dominant genus is *Lactobacillus* (45.81–99.00%), which is consistent with the previous report on koumiss (23). There are more species of bacteria from Q and Z regions, and their bacterial composition is more complex. Compared with other areas, the Z region contains a certain abundance of *Lactococcus* (11.40–17.98%), which may be related to the climate and proximity to the city. There are significant differences in the relative abundance of bacterial genera in Hurunge from different regions. *Acetobacter* (0.08–22.41%) is the second dominant genus. The contents of BEG, HHA, QHA, and ZY fermented in the capsule are significantly higher than those in the traditional fermentation method. In the sample QHA, *komagataeibacter* exists uniquely, and the content is as high as 26.74%. This genus is a new genus isolated from *Acetobacter* in 2012 (24).

### Functional Prediction of Bacterial Community

**Figure 6:**
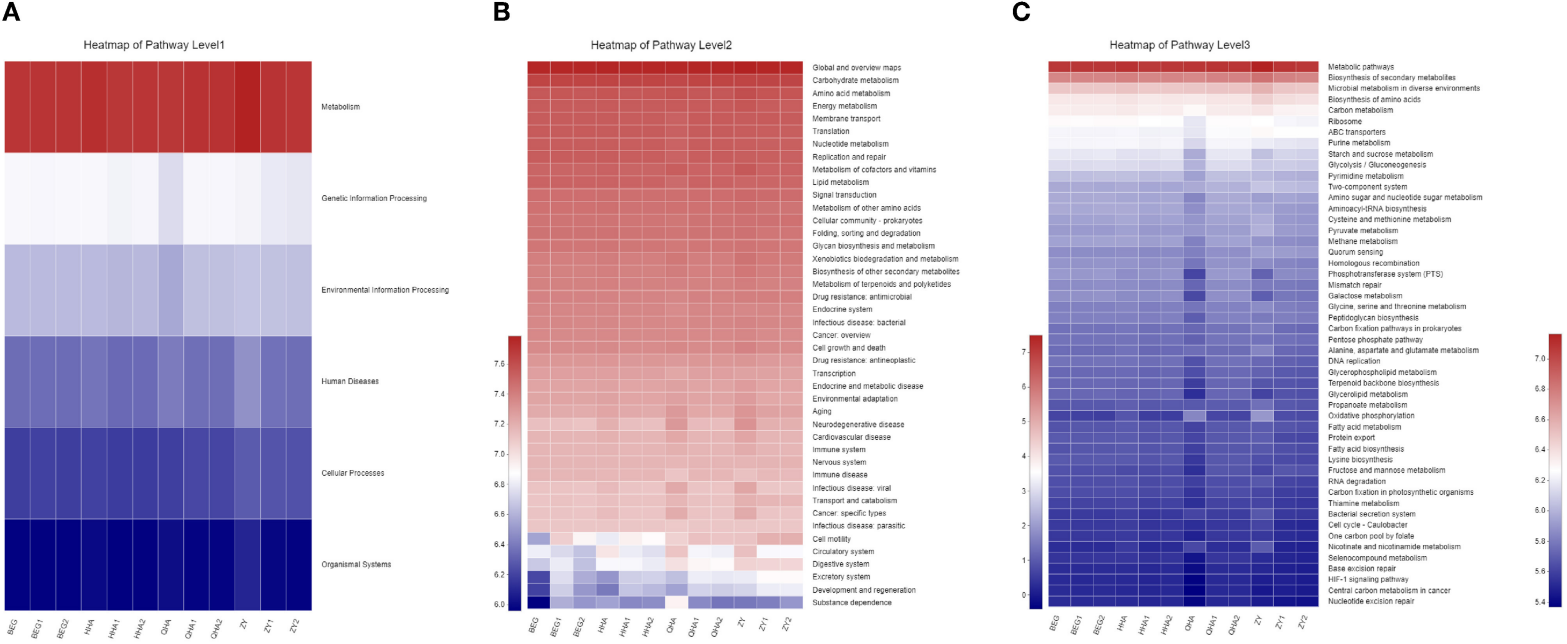
Prediction of the bacterial community in different koumiss samples. **(A)** Overall KEGG gene function statistics (Level 1). **(B)** The heatmap of functional pathways (Level 2). **(C)** The heatmap of functional pathways (Level 3).

The KEGG PATHWAY analysis was used to predict the biological functions of bacteria in six areas: metabolism, genetic information processing, environmental information processing, cell processes, human diseases, and organism systems. As shown in Figure 6A, the abundance of metabolism-related genes is the highest in all samples, indicating that metabolism plays an important role in the microbial community and that the rapid growth of microorganisms is closely related to the abundance of metabolic genes.

The level 2 KEGG PATHWAY analysis is shown in Figure 6B. The color depth of each module in the functional heat map represents the different richness of each group of functional genes. Genes related to carbohydrate and amino acid metabolism exist and are highly rich in all populations, which highlights the biological significance of functions such as metabolism and rapid growth of microorganisms. The decomposition of carbohydrates usually acts as an energy source for the growth and development of microorganisms in the process of dairy fermentation (25), and amino acid catabolism is the core function of *bacteria*. Various bacteria play an important role in providing nutrition to other microorganisms in “Hurunge” by decomposing proteins into small molecules of amino acids (26). In addition, nucleotide metabolism, energy metabolism, cofactor and vitamin metabolism, and lipid metabolism pathway are also very rich in each group of samples. Especially in QHA and ZY samples, all the metabolic functions have a relatively high abundance. However, some genes related to human diseases, such as drug resistance, antibiotics, infectious diseases, cancer, and immune diseases, have been annotated. The existence of these genes might have adverse effects on human health.

The results of the grade 3 KEGG heat map, as shown in Figure 6C, show that the gene abundance of the metabolic pathway is the highest. The annotated results reveal the potential advantages of microorganisms in traditional fermented “Hurunge,” which contain genes involved in a large number of metabolic processes, which are involved in important life pathways such as glucose metabolism and lipid metabolism. The abundance of genes such as biosynthesis of secondary metabolites, microbial metabolism in different environments, and biosynthesis of amino acids are at the forefront.

## Discussion

High-throughput sequencing has promoted significant progress in the understanding of microbial ecology. It is now widely used in many fields, from personalized medicine (27) to bioenergy (28). By sequencing the bacterial 16S rRNA gene, the community structure of microorganisms in the sample can be effectively revealed. However, so far, the microbial diversity of Hurunge samples in different regions of Mongolia has not been reported. Therefore, the bacterial diversity of 12 Hurunge samples collected from Bulgan Province, Ovorkhangay Province, Arkhangay Province, and Tov Province of Mongolia was studied by high-throughput sequencing of the 16Sr RNA gene. Hurunge is very important for brewing delicious koumiss in the coming year, and it is also the inheritance of the Mongolian working people to the excellent production technology of traditional koumiss. The regional environment, milking space environment, special fermentation mode, fermentation container, and temperature affect the richness and diversity of bacteria in Hurunge samples. Kamimura (29) showed that samples from different regions will have significant differences in bacterial flora structure due to different production environments. Samples QHA, ZY, HHA, and BEG are fermented in animal skins. The fermentation method is to put fresh horse milk in animal skins containing a small amount of fermented koumiss. In hot summer, it is usually hung on the horse's back. With the horses running, the horse milk in the skins is fully stirred to accelerate the fermentation of sour horse milk. The fermentation environment is a relatively closed small container sewn with animal skins. Therefore, compared with Hurunge made by traditional natural fermentation, the richness, and diversity of bacteria are low, but the microbial lineage contained in Hurunge in different regions is relatively stable.

PCoA and sample level cluster analysis showed the relationship between microbial diversity in the four regions and further emphasized that there were differences in microbial diversity among different regions, and they were related to geographical location. Vegetation in Mongolia ranges from the forest through grasslands to the sparse vegetation of the Gobi Desert from north to south, with steppe grasslands accounting for nearly 88% of the total land area (30). Area Z is located in the north of Mongolia, with precipitation decreasing from north to south, and water vapor mainly comes from the Arctic Ocean. Due to low temperature, less evaporation, and relatively humid climate, it should be a semi-humid area in climate zoning. It is found that relative humidity is the most important factor affecting the relative abundance of microbial communities (31). Region Z is relatively humid compared with regions B, H, and Q, so region Z is far away from the other 3 regions on the PCoA map. Another reason may be that area Z is located around the city, while areas B, H, and Q are located in sparsely populated primitive areas. Therefore, it is speculated that human production activities have a certain impact on the bacterial diversity in Hurunge, and the specific influencing factors need to be further studied. Burgan province is a well-known region of Mongolia rich in high-quality mare's milk, and the quality of its brewed koumiss is self-evident, and Hurunge in this region has the highest OTU. Therefore, it is inferred that the high-quality quality of koumiss in this region is closely related to the richness of microorganisms.

At the phylum level, *Firmicutes* is dominant in all 4 regions, followed by *Proteobacteria*, which is consistent with the results of Guo et al. (6) on the dominant bacteria in koumiss in Inner Mongolia. It may be that bacterial diversity is affected by geographical location, climatic conditions, environment, and animal feed (32); Hurunge's production methods also vary from region to region (33–35). For example, in Burgan Province, which is famous for its taste, they use the unique fermentation method invented by themselves to put Hurunge into the leather bag containing animals and hang it on the horse's back in hot weather. That is, with the running of the horse, the temperature rises and the fermentation speeds up so that the quality of koumiss is delicious.

Lactic acid bacteria are the key bacteria in the fermentation process. The content of lactose in fresh mare's milk is high. Lactic acid bacteria can use the disaccharide in lactose for fermentation, hydrolyze protein and fat to a certain extent, help to improve the taste and aroma of fermented milk (36), and then facilitate human digestion and nutrient absorption. It is for this reason that patients with lactose intolerance can drink koumiss. It is also believed that the type of bacteria in the final fermentation product is related to its acid resistance. Generally speaking, the acid resistance of *Lactobacillus* is higher than that of *Lactococcus*, because *lactobacillus* has an unknown tolerance mechanism. The acidity of koumiss after fermentation will be very high, which might be one of the important reasons why lactobacillus is a dominant bacterium (37). This is also the reason why *lactobacillus* is the dominant genus and has a high abundance in all 4 regions. The interaction of microorganisms in the system forms the unique taste and texture of koumiss wine. The *Lactobacillus* content of fermented samples stored in animal skin bags is lower than that of ordinary fermentation methods, and the content of *Acetobacter* is higher. Because *Acetobacter* contains alcohol dehydrogenase, it can oxidize ethanol into acetic acid (38), so acetic acid bacteria can convert alcohol produced by yeast into acetic acid, and the acidity of koumiss will reach very high. Therefore, it is inferred that koumiss fermented in skin bags, the acidity is higher than that of traditional fermented koumiss.

In the sample QHA, a special bacterium *komagataeibacter* was found, which has a strong ability to produce bacterial nano cellulose. This substance has long-term application prospects in the fields of cosmetics, composite materials, and wound care (39). Khan, H isolated a *komagataeibacter xylinus* IITR DKH20 from rotten apples, improve the yield of bacterial nano cellulose by optimizing the culture medium (40); Top, B isolated a *komagataeibacter xylinus* S4 (41) from homemade wine vinegar; Gopu, G isolated a *komagataeibacter sacharivorans* strain BC1 from rotten green grapes (42). However, it has been reported that *komagataeibacter* was found and isolated from the safe traditional fermented koumiss, which shows that the bacteria of this genus have strong acid resistance, which provides a new research direction for the separation of bacteria producing nano bacterial cellulose in the future.

However, we found traces of foodborne pathogens in all four regions, such as *Enterbacter, Citrobacter*, and other pathogenic bacteria, which were not detected in previous studies (43). *Enterbacter* is a natural resident of the human and animal gastrointestinal tract. It also exists in vegetables, raw meat, milk, and cheese (44). Luoyizha et al. (45) found that *Enterobacter* in raw donkey milk does not pose a risk to human health and can be explored as a potential starter/food fermentation auxiliary culture. Therefore, it is speculated that the *Enterobacter* in the Hurunge sample may not cause harm to the human body. The existence of *Enterobacter* maybe because the koumiss is usually produced by the open natural fermentation process, and there may be bacterial pollution in the initial raw milk. Therefore, in the production process of traditional fermented koumiss, while improving the sanitary environment, we should gradually transition from traditional production to modern standardized production, which will help to reduce the pathogens in the environment. These results suggest that the structural differences of bacterial communities may be related to geographical location. In addition, since many bacteria have not been identified at the species level and a few have not been detected, it is possible to identify new bacteria in Hurunge, which will provide new ideas and directions for future research.

The KEGG database is a large knowledge base for systematically analyzing gene functions and connecting genomic information and functional information. The KEGG PATHWAY database includes various metabolic pathways, synthetic pathways, membrane transport, signal transmission, cell cycle, and disease-related pathways. In addition, it also collects various chemical molecules enzyme and enzymatic reactions (46). Through the functional prediction of the bacterial community, the potential risk of bacteria carrying pathogenic genes in samples can be further evaluated to ensure the edible safety of different dairy products. However, the metabolic function predicted by the KEGG PATHWAY in this study is the basic metabolic function of microorganisms, which has a certain error. It is necessary to further confirm the metabolism of each sample at the genome level through metagenomic sequencing (47).

## Conclusion

With the increasing variety of portable and convenient foods and the continuous infiltration of modern lifestyle into pastoral areas, the types and processing capacity of Hurunge continue to decrease, and the microbial resources contained therein gradually disappear. Therefore, in this study, 12 precious Hurunge samples collected from four regions of Mongolia were used to analyze the diversity and predict the function of bacterial communities based on 16S rRNA. The results showed that the bacterial community structure and function of Hurunge were affected by the geographical sources and fermentation methods. Among the 12 Hurunge samples, the total number of bacterial species in sample BEG1 is the largest, and the diversity of the bacterial community in sample ZY2 is the highest. A total of 120 species in 98 genera and 17 phyla are identified, of which *Firmicutes* and *Lactobacillus* are the dominant phylum and genus, respectively, *Acetobacter* is the second dominant genus, and the sample QHA contains *komagataeibacter* with the potential ability to produce bacterial nano cellulose. Compared with traditional fermentation methods, Hurunge samples BEG, HHA, QHA, and ZY fermented by animal skin such as fermentation have lower OTU levels, higher *Acetobacter* content, and different bacterial diversity. The abundance of *Lactococcus* in the Z region is significantly higher than that in the other three regions. The KEGG function prediction analysis shows that new and old metabolism occupies a dominant position in all microorganisms, this is conducive to the rapid growth and reproduction of microorganisms. The high abundance of carbohydrate and amino acid catabolic genes are closely related to the flavor, texture, and final product function of koumiss. The purpose of this study is to analyze the bacterial diversity of Hurunge collected from Mongolia, so as to lay a foundation for the isolation and identification of probiotics and the protection of microbial resources in Hurunge in the future. At the same time, it is also providing the necessary theoretical basis for the production of koumiss by Hurunge at the factory level, which is more conducive to the development of probiotics and carries forward traditional national dairy products.

## Data Availability Statement

The datasets presented in this study can be found in online repositories. The names of the repository/repositories and accession number(s) can be found below: SRA, PRJNA793521.

## Author Contributions

WB, YH, and WL designed the experiments and conducted most of the experiments. WB and YH wrote and edited the manuscript. WB and WL reviewed the manuscript. All authors read and approved the manuscript.

## Funding

This study was supported by grants from the Transformation Program of Scientific and Technological Achievements of Inner Mongolia (2020CG0012), Intergovernmental Key Project for International Cooperation in Science, Technology and Innovation (2017YFE0108700), and Scientific Research Program of Colleges and Universities in the Inner Mongolia Autonomous Region (NJZY19054).

## Conflict of Interest

The authors declare that the research was conducted in the absence of any commercial or financial relationships that could be construed as a potential conflict of interest.

## Publisher's Note

All claims expressed in this article are solely those of the authors and do not necessarily represent those of their affiliated organizations, or those of the publisher, the editors and the reviewers. Any product that may be evaluated in this article, or claim that may be made by its manufacturer, is not guaranteed or endorsed by the publisher.
